# Revising the lower statistical limit of x-ray grating-based phase-contrast computed tomography

**DOI:** 10.1371/journal.pone.0184217

**Published:** 2017-09-06

**Authors:** Mathias Marschner, Lorenz Birnbacher, Marian Willner, Michael Chabior, Julia Herzen, Peter B. Noël, Franz Pfeiffer

**Affiliations:** 1 Chair of Biomedical Physics, Department of Physics and Munich School of BioEngineering, Technical University of Munich, 85748 Garching, Germany; 2 Department of Diagnostic and Interventional Radiology, Klinikum rechts der Isar, Technical University of Munich, 81675 München, Germany; 3 Institute for Advanced Study, Technical University of Munich, 85748 Garching, Germany; Lunds Universitet, SWEDEN

## Abstract

Phase-contrast x-ray computed tomography (PCCT) is currently investigated as an interesting extension of conventional CT, providing high soft-tissue contrast even if examining weakly absorbing specimen. Until now, the potential for dose reduction was thought to be limited compared to attenuation CT, since meaningful phase retrieval fails for scans with very low photon counts when using the conventional phase retrieval method via phase stepping. In this work, we examine the statistical behaviour of the reverse projection method, an alternative phase retrieval approach and compare the results to the conventional phase retrieval technique. We investigate the noise levels in the projections as well as the image quality and quantitative accuracy of the reconstructed tomographic volumes. The results of our study show that this method performs better in a low-dose scenario than the conventional phase retrieval approach, resulting in lower noise levels, enhanced image quality and more accurate quantitative values. Overall, we demonstrate that the lower statistical limit of the phase stepping procedure as proposed by recent literature does not apply to this alternative phase retrieval technique. However, further development is necessary to overcome experimental challenges posed by this method which would enable mainstream or even clinical application of PCCT.

## Introduction

Limited soft-tissue contrast is a major limitation of x-ray computed tomography (CT), a widely used clinical imaging modality. One way to address this shortcoming is to employ phase-sensitive imaging methods, which access the real part of the refractive index of the measured object [1–7]. The refractive index decrement leads to a phase shift of x-rays when passing through an object. The cross-section of this interaction is orders of magnitude higher in comparison to the cross-section of the attenuation interaction [8]. Thus, when this information can be used for imaging, increased contrast is reached.

While several phase-sensitive imaging techniques exist, only some yield favourable preconditions for a laboratory implementation [4, 7–11]. One promising technique for tomographic imaging with laboratory sources is called x-ray grating interferometry. In this approach, x-ray gratings are used to encode phase information in sinusoidal intensity variations of a series of multiple image frames. The phase information can be retrieved by e.g. Fourier analysis of these so-called phase-stepping curves. Several studies employing this technique have been published, which highlight its potential diagnostic benefits compared to attenuation-based CT [12, 13]. Moreover, the first radiographic and tomographic studies on living small animals were demonstrated [14, 15]. Additionally, this method has lately received increasing interest from manufacturers of medical imaging products [16, 17].

However, there are remaining difficulties which prohibit the use of PCCT in a clinical setting that have to be addressed. Among these, the reduction of scan time and a lower exposure to ionizing radiation are especially important. Over the last years, research on attenuation-based CT achieved significant improvement with regard to radiation dose reduction [18–21], suggesting the same possibilities for phase-contrast CT (PCCT). Yet, this claim was disputed in recent literature [22]. They state that a minimum radiation dose per projection for PCCT exists when phase retrieval based on Fourier analysis is used. This low-dose limit is a direct consequence of a phenomenon called statistical phase wrapping. This effect occurs at low photon counts and has negative effects on the image quality. In particular, statistical phase wrapping causes a drastic increase in image noise and leads to incorrectly measured phase shifts. Therefore, alternative methods to extract the phase information are to be examined that may be able to extract the phase information correctly even in the case of very low photon counts.

Lately, an alternative phase retrieval scheme, the reverse projection (RP) approach, has been adopted from diffraction enhanced imaging [23]. In this approach, the phase shift of the sample is obtained using only two phase steps and a linear approximation of the sinusoidal phase stepping curve [24]. Since this method avoids non-linearity in the phase retrieval, it is expected to have a more favourable behaviour at low photon counts. The noise properties of this novel technique compared to the phase stepping procedure have been investigated analytically as well as through simulation studies [25, 26]. However, these studies cover only the case of high photon statistics or low noise, where Fourier-based phase retrieval still produces reliable results. Correspondingly, a closer look at the behaviour of the reverse projection method at low photon counts is necessary. Especially two important properties need to be investigated in the low-dose regime: the amount of noise in the retrieved phase-contrast images as well as whether the measured values are quantitatively correct. Given these properties, the RP method could be able to solve the problem of collapsing signal propagation that occurs in the methods based on Fourier analysis.

In this article, we examine the statistical properties of the RP phase retrieval method at low photon counts and compare it to the widely-used phase retrieval method based on Fourier analysis. We study experimentally the dependence of the standard deviation of the retrieved phase values on the number of photons per pixel, evaluated over an empty region in the differential phase-contrast projections and in the phase stepping images. Additionally, we present first tomographic results obtained with the RP technique using a Talbot-Lau interferometer with a laboratory x-ray tube. We use these results to first compare the image quality of these tomographic images with those obtained using the Fourier-based method. We further illustrate how the image quality of the tomographic measurements is affected by statistical phase wrapping in the projections when employing the Fourier-based approach. Second, we assess the correctness of the quantitative values obtained by the two phase retrieval methods for the cases of high and low photon counts. Finally, we discuss the implications of the results for the purpose of low-dose phase contrast CT imaging.

## Materials and methods

The study was approved by the institutional review board (Technische Universität München, Klinikum rechts der Isar, Munich, Germany).

### Grating-based phase-contrast imaging

In grating-based phase-contrast imaging, x-ray gratings are used to obtain information on the refraction and scattering properties of the sample. Three imaging modalities are simultaneously retrieved: first, the attenuation of the sample, which is equivalent to the signal obtained in conventional radiography. Second, the differential phase shift, which is related to the refractive index of the sample and proportional to its electron density. And third, the so-called dark-field signal, which arises from small angle scattering. A detailed description of x-ray grating interferometry can be found elsewhere [4, 8–10].

A so-called Talbot-Lau interferometer is used when employing a conventional laboratory x-ray tube with insufficient spatial coherence [9, 10]. This kind of interferometer, which was employed in this work, consists of three gratings. The first grating (G_0_) is an absorbing grating, which is placed directly behind the source and provides transverse beam coherence. The second grating (G_1_), which is placed close to the sample to enhance sensitivity, is a phase grating that imposes a periodic phase modulation onto the x-ray beam. This creates an interference pattern at certain distances downstream, the so-called fractional Talbot distances. A phase shifting (refractive) sample in the beam leads to a displacement of the interference pattern. This pattern cannot be resolved directly because the period of the gratings and thus the period of the interference pattern is much smaller than the pixel size of conventional detectors. Small grating periods enable high sensitivity with compact setup geometries. Therefore, a third grating (G_2_), which is an absorbing grating with a period matching the intensity pattern, is installed to analyse this pattern.

### Phase stepping procedure and phase retrieval

To analyse the intensity pattern generated by the phase grating and the sample, one of the gratings needs to be translated. That means that the sample is recorded multiple times with different relative grating positions. These images can be combined to phase stepping curves for each pixel, which are of sinusoidal shape [4, 11]. The information related to absorption, refraction and scattering strength can then be extracted by Fourier analysis or least-squares fit of these curves. In particular, a map of the phase shift of these curves corresponds to the differential phase-contrast projection, which is called *φ*(*x*, *y*) with pixel positions x and y. Generally, the differential phase recorded at the detector is not constant across the entire image, as it is the case with the absorption image as well. To address this issue, an additional projection is recorded without sample in the beam. This reference projection *φ*^*r*^(*x*, *y*) is then subtracted from the sample projection *φ*^*s*^(*x*, *y*) to obtain the differential phase shift of the sample, Δ*φ*(*x*, *y*) = *φ*^*s*^(*x*, *y*) − *φ*^*r*^(*x*, *y*). The method of using a Fourier analysis or least-squares fit of a complete stepping curve to retrieve the phase shifts is referred to as the phase stepping (PS) approach in this article.

In order to comply with clinical boundary conditions, the exposure time or the radiation dose that can be applied to obtain one projection is limited. This constraint implies the necessity to reduce the number of phase steps per projection or to reduce the exposure time per phase step. The former is possible down to a minimum of three phase steps that are necessary to reconstruct the sinusoidal phase stepping curve. The latter entails more noise in the acquired stepping images, which also leads to more noise in the differential phase-contrast projections. The noise propagation of this kind of phase retrieval method has been studied in detail [27–29]. Two separate cases have to be considered, since the noise properties change when approaching the limit of low photon counts.

In the case of high statistics i.e. high photon counts and sufficient visibility, the standard deviation of the retrieved phase in the differential phase-contrast (DPC) projections is proportional to the standard deviation of the measured photon counts in the phase stepping images *σ*_*L*_. This standard deviation is itself proportional to the inverse square root of the number of photon counts, assuming the noise is created only by Poisson statistics using a photon counting detector. Explicitly, the standard deviation in the DPC projections is given by

$$\begin{array}{c} \sigma_{\varphi }=\frac{\sqrt{2}}{v}\sigma_{L}=\frac{\sqrt{2}}{v}\frac{1}{\sqrt{NI}}\;. \end{array}$$ (1)

Here, the number of quanta per pixel and phase step is called *I*, while *N* stands for the number of phase steps. The visibility *v* of the interferometer is a measure of the amplitude of the intensity oscillation for each pixel and is calculated by the ratio of the first and the zero-order Fourier coefficient of the phase stepping curve of the reference scan.

In the case of low photon counts per pixel, the stochastic properties change. Since the measured differential phase is non-ambiguous only in the interval *I*_*φ*_ = ]−*π*, *π*[, problems arise when the standard deviation is high and the distribution function of the differential phase *φ* has non-negligible values at the boundaries of this interval. Then, Gaussian error propagation is no longer valid and some pixels can be affected by statistical phase wrapping [22]. In these pixels, the error in the phase retrieval that is caused by the noise in the phase stepping images leads to the effect that the phase wraps around the borders of the interval *I*_*φ*_ and a wrong value is measured. When decreasing the exposure time, more and more pixels are affected by statistical phase wrapping. This leads to a faster increase of *σ*_*φ*_, the standard deviation in the DPC projections, than the purely Poisson-based theory would suggest. Note that this effect is different from ‘normal’ phase wrapping that stems from large phase shifts of the sample itself and which is independent from the measurement procedure and exposure time.

In the low-count limit *σ*_*L*_ → ∞, the standard deviation converges towards $\sigma_{\varphi }=\pi /\sqrt{3}\approx 1.81$, which is the standard deviation of the uniform distribution on the interval *I*_*φ*_ [22]. Although the standard deviation rises only to a lesser degree when reducing the number of photons in this region, this does not correspond to an improved image quality. Quite the contrary, it represents the complete collapse of signal transmission for all pixels, which means that the retrieved phase shift contains no useful information any more. In conclusion, statistical phase wrapping entails that the phase retrieval does not provide reliable results in affected pixels, which corresponds to a collapse of information transmission. Therefore, a lower limit for the number of photons, i.e. the applied radiation dose exists for successful phase retrieval when using a phase stepping procedure.

### Alternative phase retrieval through linear approximation

The reverse projection method is an alternative method of phase retrieval [24]. In this approach, the sinusoidal phase stepping curve is approximated at its steepest points by a linear function. Instead of performing a complete phase stepping scan, only two projections at selected grating positions are recorded. This method has been extended to also work with 2-dimensional gratings [30] and fan-beam geometry [31]. In the following, it is reviewed shortly.

In grating-based phase-contrast imaging, the intensity recorded at each detector pixel and grating position can be expressed by

$$\begin{array}{c} I\left(x,y,x_{g}\right)=a_{0}\left(x,y\right)+a_{1}\left(x,y\right)\sin\left(2\pi \frac{x_{g}}{g_{2}}+\varphi \left(x,y\right)\right)\;, \end{array}$$ (2)

where *a*_0_(*x*, *y*) and *a*_1_(*x*, *y*) represent the zeroth and first Fourier coefficients of the sinusoidal stepping curve. The period of the analyser grating is called *g*_2_ while *x*_*g*_ denotes the relative transverse shift of the gratings.

The phase stepping curve has two regions where its slope is linear, provided that a phase stepping is performed over one period. There, it can be approximated with a linear function resulting in

$$\begin{array}{c} I\left(x,y,x_{g}\right)\approx a_{0}\left(x,y\right)\pm a_{1}\left(x,y\right)\varphi \left(x,y\right)\;. \end{array}$$ (3)

**Fig 1:**
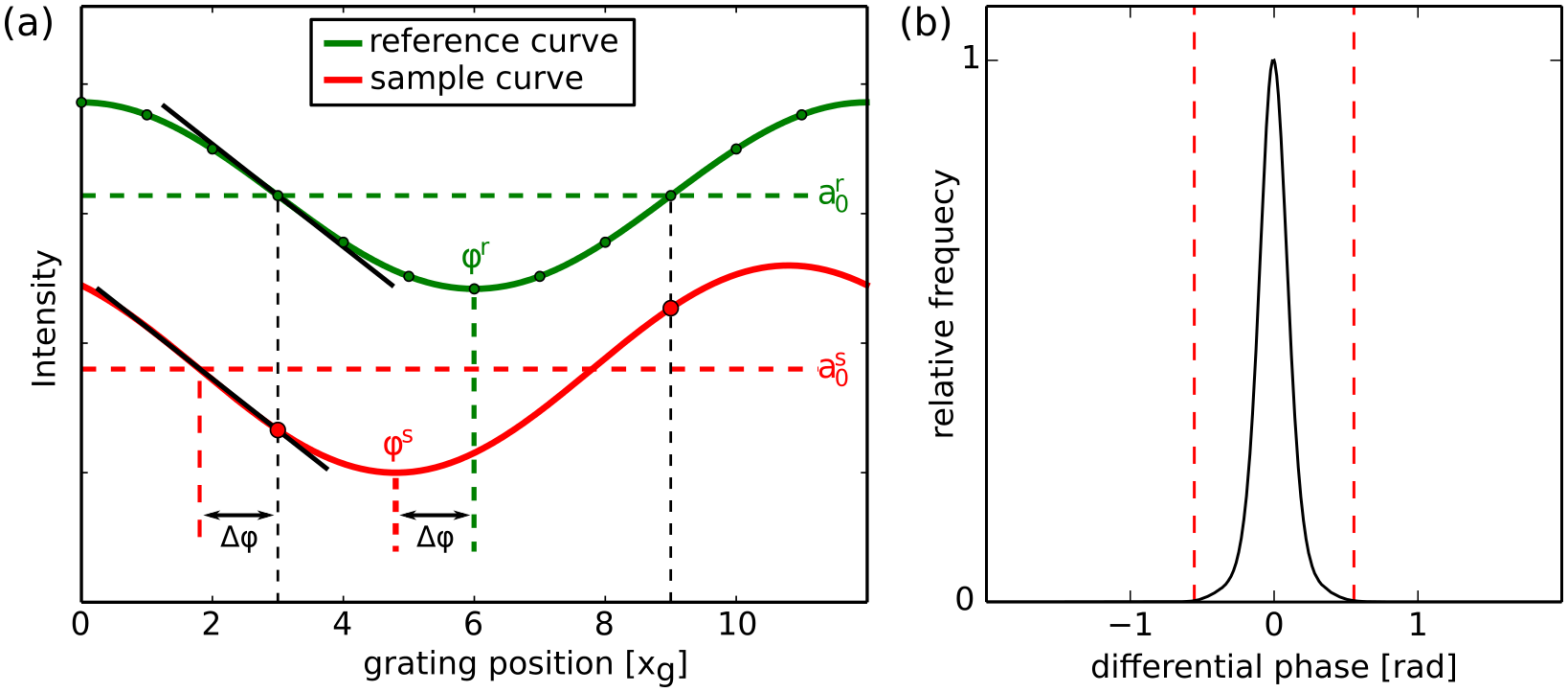
Principle of the reverse projection method. Starting from the phase stepping curve (a) that is recorded without sample (reference scan), the sample is measured with grating positions corresponding to the two linear regions of the stepping curve. The two recorded intensities can then be used to obtain the attenuation $a_{0}^{s}$ of the sample as well as its differential phase shift Δ*φ*_*s*_. Panel (b) shows the histogram of the differential phase-contrast projections of a tomographic scan of a biomedical sample. The red lines mark the region where the error of the linear approximation is less than 5%. Only 0.1% of all pixels lie outside of this region.

Exemplary phase stepping curves of a sample scan and a reference scan are displayed in Fig 1(a), where also a linear region is highlighted.

This linear approximation will now be used to determine the attenuation and phase shift of the sample. First, a reference scan is recorded featuring a full stepping curve. Then, a projection with the sample in the beam is acquired at each of the two grating positions that correspond to the linear regions of the reference scan stepping curve, i.e. where $I^{r}=a_{0}^{r}$, and thus

$$\begin{array}{c} x_{g,1}=\frac{-\varphi^{r}}{2\pi }g_{2}\;\;\;\;\text{and}\;\;\;\;x_{g,2}=\left(\frac{-\varphi^{r}}{2\pi }+\frac{1}{2}\right)g_{2}\;. \end{array}$$ (4)

The measured intensities at these gratings positions are

$$\begin{array}{ccc} I_{1}\left(x,y\right) & = & I(x,y,x_{g,1})=\\ & = & a_{0}^{s}+a_{1}^{s}\sin\left(2\pi \frac{x_{g,1}}{g_{2}}+\varphi^{s}\left(x,y\right)\right)=\\ & = & a_{0}^{s}+a_{1}^{s}\sin\left(2\pi \frac{x_{g,1}}{g_{2}}+\Delta \varphi \left(x,y\right)+\varphi^{r}\left(x,y\right)\right)=\\ & = & a_{0}^{s}+a_{1}^{s}\sin\left(\Delta \varphi \left(x,y\right)\right)\approx \\ & \approx & a_{0}^{s}\left(x,y\right)+a_{1}^{s}\left(x,y\right)\Delta \varphi \left(x,y\right)\; \end{array}$$ (5)

and equivalently

$$\begin{array}{ccc} I_{2}\left(x,y\right) & = & I(x,y,x_{g,2})=\\ & \approx & a_{0}^{s}\left(x,y\right)-a_{1}^{s}\left(x,y\right)\Delta \varphi \left(x,y\right)\;. \end{array}$$ (6)

These projections can be combined to obtain the attenuation and differential phase-contrast projections. The attenuation signal can be determined by taking the mean value of the two measurements and normalizing it to the average intensity in the reference image $I_{0}=a_{0}^{r}$, as shown in the following expression

$$\begin{array}{c} a\left(x,y,\theta \right)=\frac{a_{0}^{s}}{a_{0}^{r}}=\frac{I_{1}\left(x,y\right)+I_{2}\left(x,y\right)}{2a_{0}^{r}}\;. \end{array}$$ (7)

The phase shift of the stepping curve, i.e. the differential phase-contrast signal can be calculated by subtracting Eqs (5) and (6), resulting in

$$\begin{array}{c} I_{1}\left(x,y\right)-I_{2}\left(x,y\right)=2a_{1}^{s}\left(x,y\right)\Delta \varphi \left(x,y\right)\;. \end{array}$$ (8)

The slope of the linear function is determined by the first Fourier coefficient $a_{1}^{s}\left(x,y\right)$ of the sample scan. Since no phase stepping is performed for the sample scan, this coefficient is not known. However, a full phase stepping is performed for the reference scan, which is the scan without sample in the beam. This stepping curve can be used to extract $a_{1}^{r}\left(x,y\right)$, the first Fourier coefficient of the reference scan. If there is no small angle scattering i.e. no dark-field signal in the sample, these coefficients can be related to each other via the visibility, which is constant in this case. The visibility of the interferometer can be expressed by the ratio of first and zero order Fourier coefficient, namely

$$\begin{array}{c} v=\frac{a_{1}^{r}}{a_{0}^{r}}\overset{\text{no}\;\text{dark-field}}{=}\frac{a_{1}^{s}}{a_{0}^{s}}\;. \end{array}$$ (9)

Through rearranging of Eq (8) and substituting $a_{1}^{s}=v\times a_{0}^{s}$, making use of Eq (9), one arrives at

$$\begin{array}{c} \Delta \varphi \left(x,y\right)=\varphi^{s}\left(x,y\right)-\varphi^{r}\left(x,y\right)=\frac{1}{v}\frac{I_{1}\left(x,y\right)-I_{2}\left(x,y\right)}{2a_{0}^{s}}=\frac{1}{v}\frac{I_{1}\left(x,y\right)-I_{2}\left(x,y\right)}{I_{1}\left(x,y\right)+I_{2}\left(x,y\right)}\;, \end{array}$$ (10)

the equation for the differential phase-contrast signal [24].

This approximation is only valid for small values of the differential phase shift of the sample Δ*φ*(*x*, *y*). While most of the pixels are expected to be close to zero due to the differential nature of the phase-contrast signal, there are also larger values e.g. at borders between materials. These values strongly influence the quantitative values in the reconstructed volumes due to the integration step that is inherent to the reconstruction of differential values. Therefore, it is necessary to examine the accuracy of the quantitative values in the reconstructed volumes obtained with the reverse projection method. Additionally, non-negligible dark-field signal in the sample leads to wrongly determined phase shifts in the reverse projection method. It was shown recently that the retrieved signal then is the product of the objects scattering (dark-field) and phase shift signals. Thus, the phase shift is systematically underestimated in the presence of scattering in the sample [32].

Equivalently to a phase stepping acquisition, a set of projections obtained with the RP method at different tomographic angles can be used to obtain the maps of the linear attenuation coefficient *μ*(*x*, *y*, *z*) as well as the refractive index decrement *δ*(*x*, *y*, *z*). Note that the reverse projection method was implemented slightly different in the original publication [24]. There, the two projections were obtained with only one fixed grating position combined with a full scan over 360 degrees. Opposing projections can then be combined to extract the attenuation and differential phase-contrast information. This is possible since the attenuation is symmetric with rotation while the refraction is antisymmetric. In this work, we use two projections at the same tomographic angle but different grating positions for phase retrieval. For simplicity, we will still call it reverse projection method and refrain from introducing a new name here. After all, the phase retrieval algorithm is the same in both approaches and the results of this work are also applicable to the original reverse projection method. The only practical aspect that needs to be considered is that the original reverse projection method is not applicable to cone beam geometries, while our approach loses the advantages of stationary gratings.

There is one major drawback of this method, which could limit its application: To be able to acquire the two projections at the linear regions of the phase stepping curve, the phase of the reference image has to be constant over the field of view. This limitation will later be discussed in more detail.

The noise properties of the reverse projection method differ from those of the phase stepping method [25, 26]. First of all, the standard deviation of the differential phase-contrast projections is lower by a factor of $\sqrt{2}$ in the RP method. This can be explained by the fact that the region around the zero-crossings of the sinusoidal phase stepping curve is most sensitive to phase shifts. In contrast, the region around the extrema of the stepping curve is only sensitive to changes in the amplitude of the curve, i.e. to the dark-field signal. This entails that in a phase stepping approach only half of the measured points contribute to the phase information while the other half determines the dark-field signal. In the RP approach, only the points in the linear region are used, which contribute most to the phase signal. Simultaneously, no information about the dark-field signal is obtained. Therefore, only half of the data points are needed for the same precision in the phase-contrast channel. This results in a standard deviation that is lower by factor of $\sqrt{2}$ compared to the phase stepping approach when the same amount of photons are used overall [26]. Thus, the standard deviation of the retrieved differential phase contrast signal is given by [25]

$$\begin{array}{c} \sigma_{\varphi }=\frac{1}{v}\sigma_{L}=\frac{1}{v}\frac{1}{\sqrt{NI}}\;. \end{array}$$ (11)

More interestingly, a linear phase retrieval might not suffer from the same problems at low statistics as the phase stepping approach due to the fact that no periodic function has to be fitted to the data points. This means that meaningful phase retrieval should be possible even for projections with very low photon counts and consequently statistical phase wrapping would be avoided. Despite this promising prospect, the noise properties of the linear phase retrieval have not yet been investigated in this low-count case.

### Experimental setup, samples and measurements

A series of tomographic scans with varying exposure times per projection were recorded to investigate the different statistical properties of the PS and the RP method. For each tomographic scan we obtained a total of 1200 sample projection evenly spanning 360 degrees as well as 300 reference projections. A filtered backprojection with a Hilbert filter kernel was used for the reconstruction, where also the slight cone-beam geometry was considered. For each projection, 11 equidistant phase steps were performed over one grating period. The exposure time, which was evenly distributed to the phase steps, was 0.025 s to 3.6 s per phase step and corresponds to a mean of around 15 to 2273 counts per pixel, respectively. Every second step, in total 5 steps, were used to obtain the phase stepping curves in the PS approach and the three imaging signals were extracted using a least-squares fit. Thus, the exposure times ranged from 0.125 s to 18 s per projection. For the RP method, the two phase steps closest to the zero crossings of the stepping curve were selected for each pixel separately. Consequently, the exposure times ranged from 0.05 s to 7.2 s per projection, which corresponds to a mean of roughly 30 counts per pixel and 4546 counts per pixel, respectively. Note, that these exposure times are theoretical in the sense that really 11 exposures were taken but only 2 particular data points (not the same for each pixel) were used for phase retrieval. This is necessary since it was not possible to achieve a homogeneous, flat reference phase in our experiment. The reference images without the sample were recorded with 11 phase steps and a total exposure time of 39.6 s per projection. Consequently, the influence of the reference images on the noise in the final projections can be neglected.

The measurements were carried out using a Talbot-Lau interferometer [33]. It consisted of three gold-plated silicon gratings with periods of 5.4 μm. The absorption gratings *G*_0_ and *G*_2_ have heights of around 65 μm. The phase grating *G*_1_ was designed to introduce a phase shift of *π* at 27 keV and has a height of 5.2 μm. The setup was installed in a symmetric configuration with inter-grating distances of 85.7 cm. The x-rays were generated by an ENRAF Nonius rotating anode x-ray tube with a molybdenum target, which was operated at 40 kVp and 70 mA. We used a PILATUS II single photon counting detector by *Dectris Ltd.*, Switzerland, which has the advantages of a box-like point spread function and no readout noise. It features a field of view of 487 pixels x 195 pixels (horizontal x vertical) with a pixel size of 172 μm x 172 μm. The sample used in this work is an ex-vivo human coronary artery. It was measured in a Falcon tube with a diameter of 3 cm filled with formalin, which was itself put in a water bath to avoid phase wrapping artifacts [34, 35]. Therefore, all reconstructed values of the linear attenuation coefficient and the refractive index decrement are given relative to water. The sample was mounted at a position where the geometric magnification of the setup was 1.72. Therefore, the effective pixel size at the sample position was 100 μm x 100 μm. The study was approved by the institutional review board.

## Results

First of all, we evaluate a scan with long exposure, i.e. high photon counts, to test the accuracy of the RP method, thereby investigating its applicability to tomographic scans of biological soft-tissue. It is clear that the error that is introduced due to the linear approximation of the sinusoidal phase stepping curve depends on the value of the differential phase-contrast signal. Thus, the distribution of values in a typical scan is of concern with regard to the approximation’s accuracy. Fig 1(b) shows the relative frequency of occurrence of phase shift values in all of the DPC projections of the tomographic scan of a biological sample. It can be clearly seen that the values are centred around a phase shift of zero, due to the differential nature of the signal. Further, the distribution of values is quite narrow with only 0.1% of pixels having an absolute value greater than *φ*^*s*^(*x*, *y*) > 0.55 rad (marked by the red dashed lines). There, the absolute error of the linear approximation is 0.028 rad while the relative error is 5%. Note that even in a scan with a very long exposure time of 18 s per projection, the standard deviation due to Poisson noise *σ*_*φ*_ = 0.07 rad is already higher than this error. Also keep in mind that this scan has been obtained at a setup with very high sensitivity that cannot be reached with a potential clinical setup, which has to be more compact. Consequently, even the phase shifts of bigger objects could still be small enough to justify using the linear approximation.

**Fig 2:**
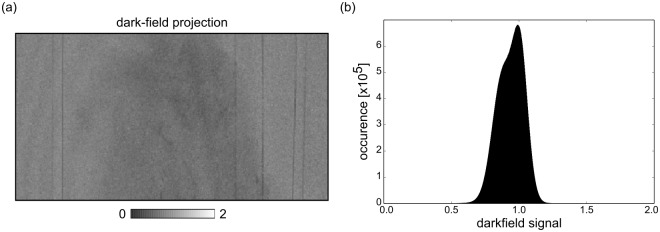
Dark-field/scattering signal strength. (a) Exemplary dark-field projection of the measured biological sample. The sample shows a smooth dark-field signal close to unity, a prerequisite for successful application of the reverse projection method. (b) Histogram of dark-field values in all projections of one tomographic scan. The peak of the sample’s dark-field is narrow and close to unity. Further, there are next to no pixels with extreme values, which could hinder the applicability of the RP method.

An additional source of error is a change in interferometer visibility due to small-angle scattering inside the sample. However, the dark-field signal of biological soft tissue is weak and is therefore not expected to significantly disturb the results obtained with the RP method (cf. Fig 2). In future work, the effect of non-negligible dark-field signal could be tackled by employing a third step [36], although the noise properties of this method have yet to be explored.

**Fig 3:**
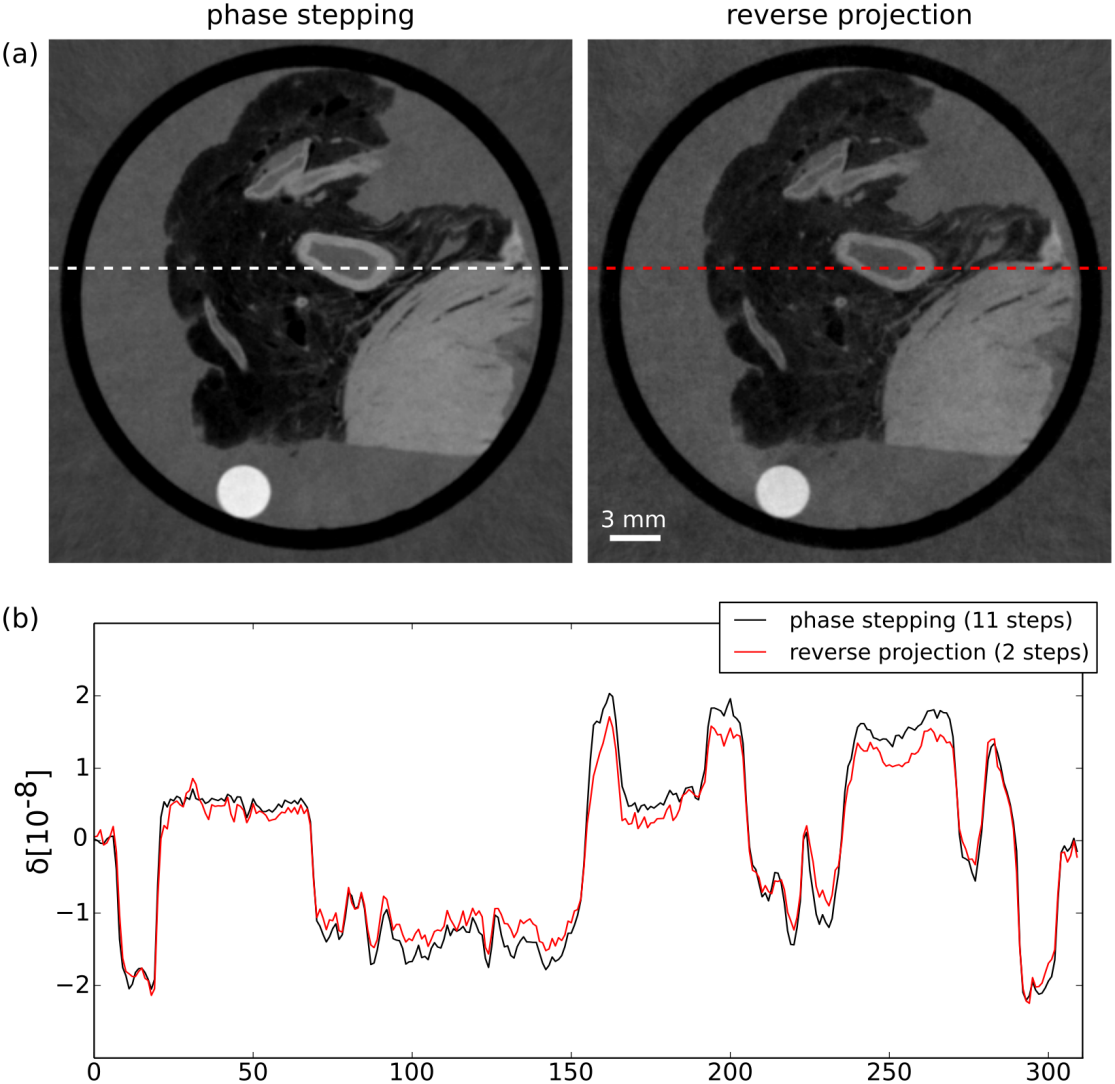
Comparing the tomographic reconstructions of a high statistic scans obtained by widely-used phase stepping approach and the reverse projection method. (a) Tomographic reconstructions of the differential phase contrast projections, obtained with the PS approach (left) and the RP method (right). (b) Line plot at the position marked by the dashed lines in (a). Both images appear very similar, which is also apparent in the line plot. The contrast in the RP image is slightly weaker, since high values are underestimated by this method.

Still using the same high-counts scan, we inspected the tomographic reconstructions of the differential phase-contrast projections gathered by the two different methods. Axial cuts of the reconstructed volume of the refractive index decrement *δ* are displayed in Fig 3(a). A visual inspection reveals no apparent morphological differences between the phase-contrast tomography slices of the two methods. However, a look at the line plot shown in Fig 3(b) reveals slight discrepancies between the two reconstructions, especially in areas with very high and very low values. In these regions, the magnitude of the refractive index decrement is underestimated in the RP reconstruction. This is an expected result since the linear approximation underestimates large phase shifts, which correspond to high absolute values of the refractive index decrement after tomographic reconstruction. In the other regions, the values are very similar for both methods, with some fluctuations due to image noise.

Additionally, the mean values in three homogeneous regions in the image volume are evaluated. The results of this analysis are displayed in rows 1 and 2 of Table 1. The differences of the quantitative values for Formalin and the Falcon tube between the two methods lie well within one standard deviation. Only for the PMMA rod, which is the bright circle visible in the reconstructed slices, the values differ more significantly: as expected, the refractive index is underestimated in the RP method by around 8% since this material exhibits a high phase shift. From this point on, the mean delta values of the PS scan with high photon counts will be used as a reference when analysing the low-counts scans.

**Table 1:** Mean values and the corresponding standard deviation of the refractive index decrement *δ* relative to water, exemplary for the materials formalin (fluid inside the tube), PMMA and the Falcon tube.

Scan desrc.	*δ*_formalin_	rel. err.	*δ*_PMMA_	rel. err.	*δ*_tube_	rel. err.
Fig 2						
reference	0.54 ± 0.14		4.65 ± 0.16		−1.94 ± 0.15	
RP (1172 cts)	0.51 ± 0.16	−5%	4.29 ± 0.19	−8%	−1.91 ± 0.17	−2%
Fig 6						
PS (70 cts)	0.37 ± 1.37	−31%	3.85 ± 1.36	−17%	−1.48 ± 1.33	−24%
RP (76 cts)	0.58 ± 0.89	7%	4.35 ± 0.86	−6%	−2.06 ± 0.81	6%
Fig 7						
PS (111 cts)	0.44 ± 1.26	−18%	3.6 ± 1.17	−23%	−1.78 ± 1.39	−8%
RP (44 cts)	0.47 ± 1.06	−14%	4.16 ± 1.05	−10%	−1.98 ± 1.03	2%

The mean values and standard deviations were evaluated over volumes containing more than 100000 voxels.

**Fig 4:**
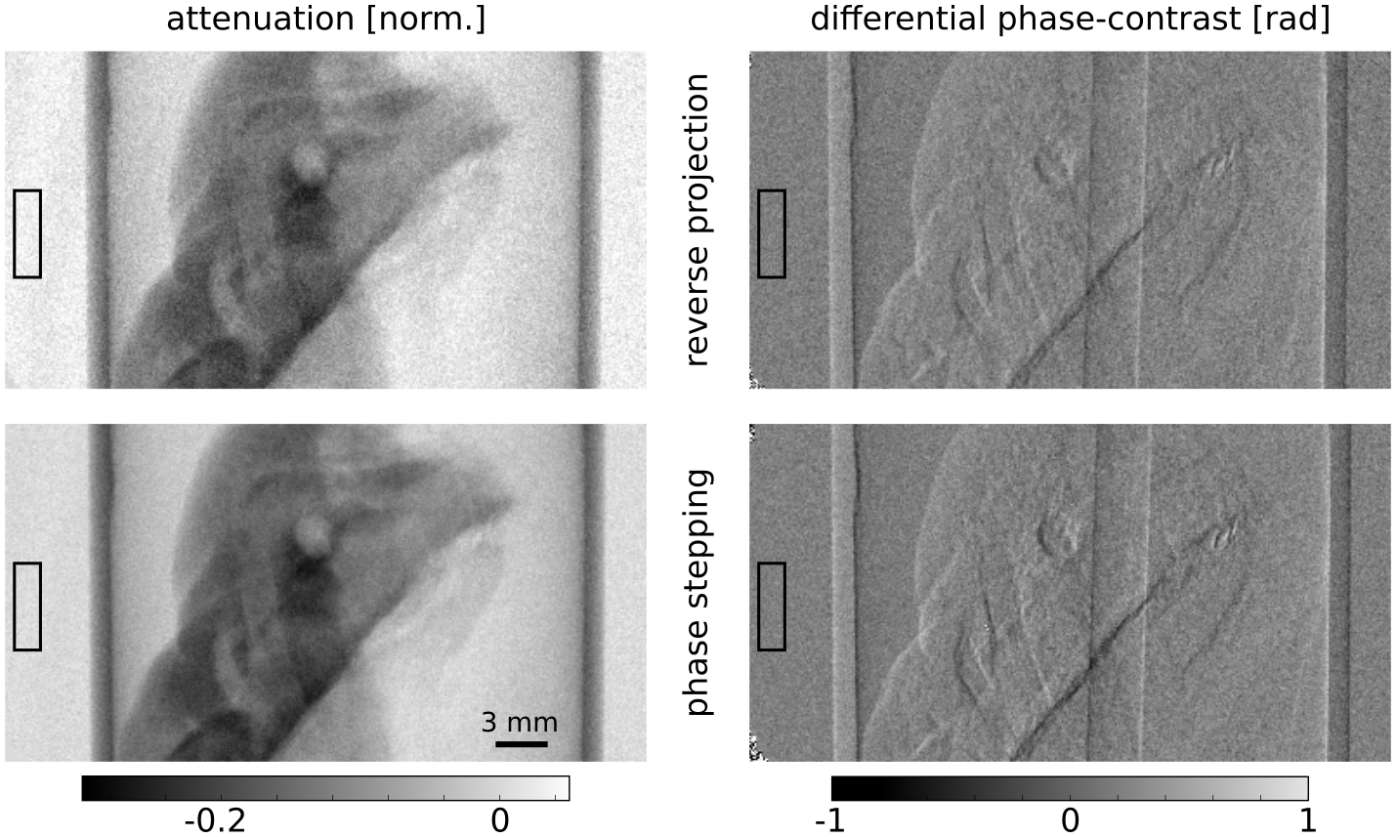
Attenuation and differential phase-contrast projections of the biological sample. The projections obtained with the reverse projection method (top) and the phase stepping procedure (bottom) overall have a very similar appearance. Difference are visible at the boarders of materials in the differential phase-contrast projection, where the RP method underestimates high values. Both projections that were obtained with the RP method show increased noise due to the fact that less steps are used there compared to the phase-stepping approach. The ROIs indicated by rectangles were used to extract the mean photon counts, standard deviation and mean visibility for the statistical analysis.

In the next step, the dependence of the image noise or the standard deviation in the differential phase-contrast projections on the exposure time or the number of photons is examined. A region of interest is defined to evaluate the number of counts in the phase stepping images, the visibility in the reference projections and the standard deviation in the DPC projections. An example of attenuation contrast and phase-contrast projections obtained with the PS procedure and the linear approximation is shown in Fig 4. Again, the visual appearance is very similar for both methods. The region of interest used for the analysis is marked by a rectangle. The mean visibility in the region of interest was measured at 18.6%.

**Fig 5:**
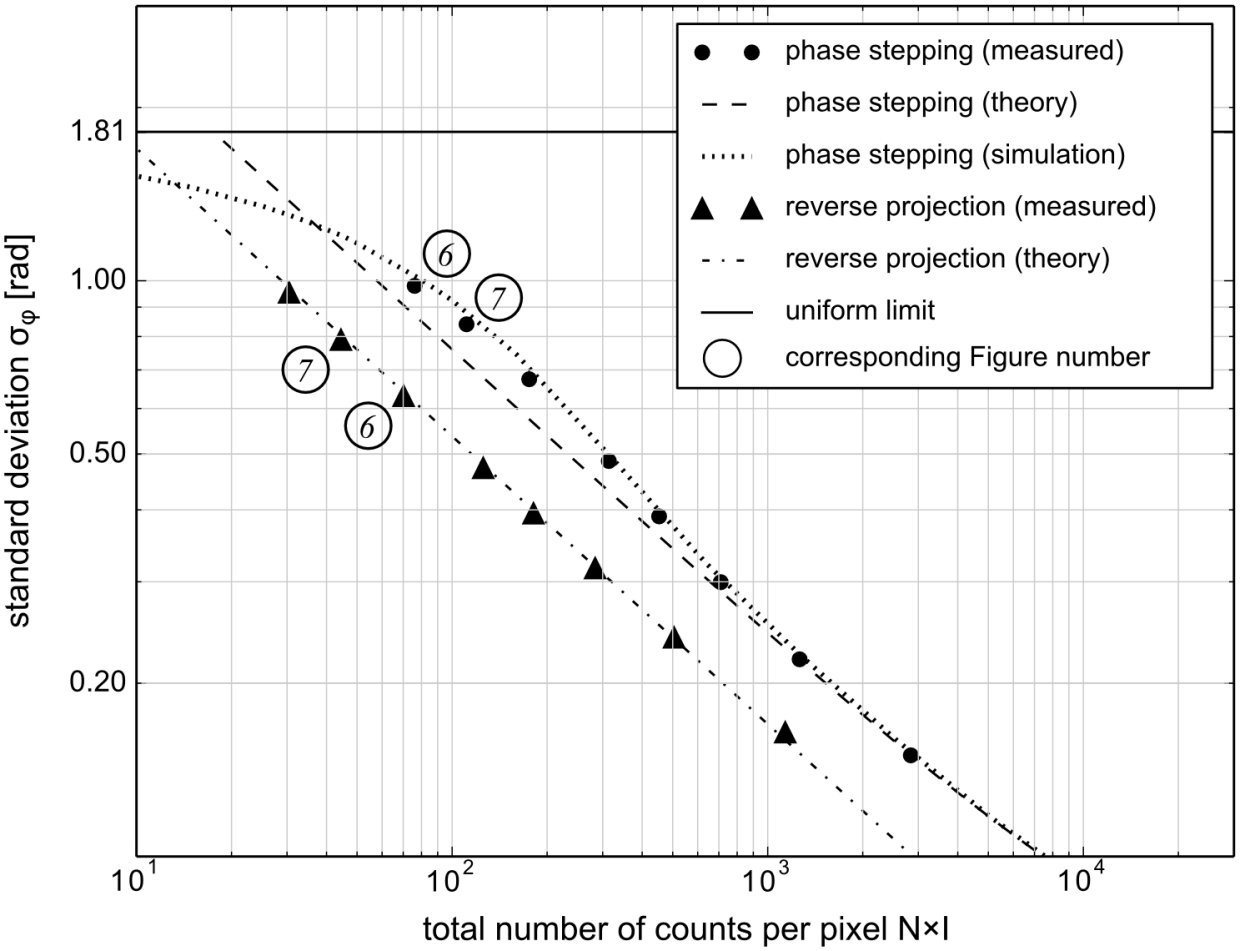
Dependency of the standard deviation of the differential phase-contrast projections on the number of photon counts per pixel. The projections obtained with the RP method show a lower standard deviation than the ones obtained with PS by a factor of $\sqrt{2}$. More importantly, in comparison to the PS procedure, the standard deviation of the RP projections does not show a deviation from Poissonian behaviour when going to lower photons counts. The circled numbers indicate the Figure in which the corresponding tomographic reconstructions are displayed.

The results of the noise analysis are shown in Fig 5, which illustrates the dependency of the standard deviation in the differential phase-contrast projections on the number of photons per pixel in the stepping images. These results will now be discussed in detail.

First of all, we evaluate the measured standard deviation of the DPC projections *σ*_*φ*_ obtained by the PS method. It can be seen that the values for projections with high photon counts agree well with the results predicted by theory [27] (cf. Eq (1)). Going towards lower mean photon counts per pixel, the standard deviation rises more rapidly than the purely Poisson-based theory suggests, which can be explained by the occurrence of statistical phase wrapping. The same behaviour is visible in the simulation results, although there the resulting values are slightly higher. This is due to the fact that the detector used in the experiment exhibits charge sharing, an effect that was not considered in the simulations. Charge sharing introduces correlation between neighbouring pixels, which in turn reduces the standard deviation slightly, especially when the mean number of photons is low.

At even lower photon counts, the standard deviation starts to saturate and is therefore getting closer again to the theoretical prediction for high statistics. This is the result of a convergence towards the uniform limit, which entails that the measured values resemble a random distribution in the interval *I*_*φ*_ = ]−*π*, *π*[ [22]. Thus, the retrieved phase is not correct any more. Therefore, scans in this regime do not show an improved image quality but instead are dominated by noise.

Next, the measured standard deviation of the DPC projections obtained with the RP method is considered. Here the theoretical values are lower by a factor of $\sqrt{2}$ compared to the PS values, as explained previously. We find that the measured values agree well with the theoretical predictions. Therefore, we have confirmed experimentally the theoretical predictions and simulations for the case of high photon counts [25, 26]. In addition to that, we could show that the standard deviation follows the theoretical values of a Poisson distribution even for scans with very low photon counts. We therefore conclude that the RP method does not suffer from statistical phase wrapping at very low photon counts, in contrast to the conventional phase retrieval technique. However, it is still to be examined whether the RP approach will also yield quantitatively correct reconstructions at these low photon counts.

For this purpose, we have compared tomographic reconstructions of the projections that were obtained with the two methods. The visual appearance of these images as well as the quantitative values of the refractive index decrement are evaluated. Additionally, the reconstructions are compared using two standard metrics: the root mean squared error (RMSE) and the structure similarity index (SSI).

First, we take a look at two scans that were acquired with nearly the same exposure time i.e. mean number of photons per pixel. In the first scan, 5 equidistant phase steps with a mean number of 15.2 counts per pixel were used to obtain the stepping curves, which was in turn analysed using a least-squares fit. For the RP scan, only 2 phase steps with a mean number of 35 counts were utilized to extract the attenuation and DPC projections. Combined, the mean number of counts per projection is then 76 per pixel for the PS and 70 per pixel for the RP method (cf. Fig 5). Filtered backprojection is once again used to reconstruct the distributions of the attenuation coefficient and the refractive index decrement from the measured projections.

**Fig 6:**
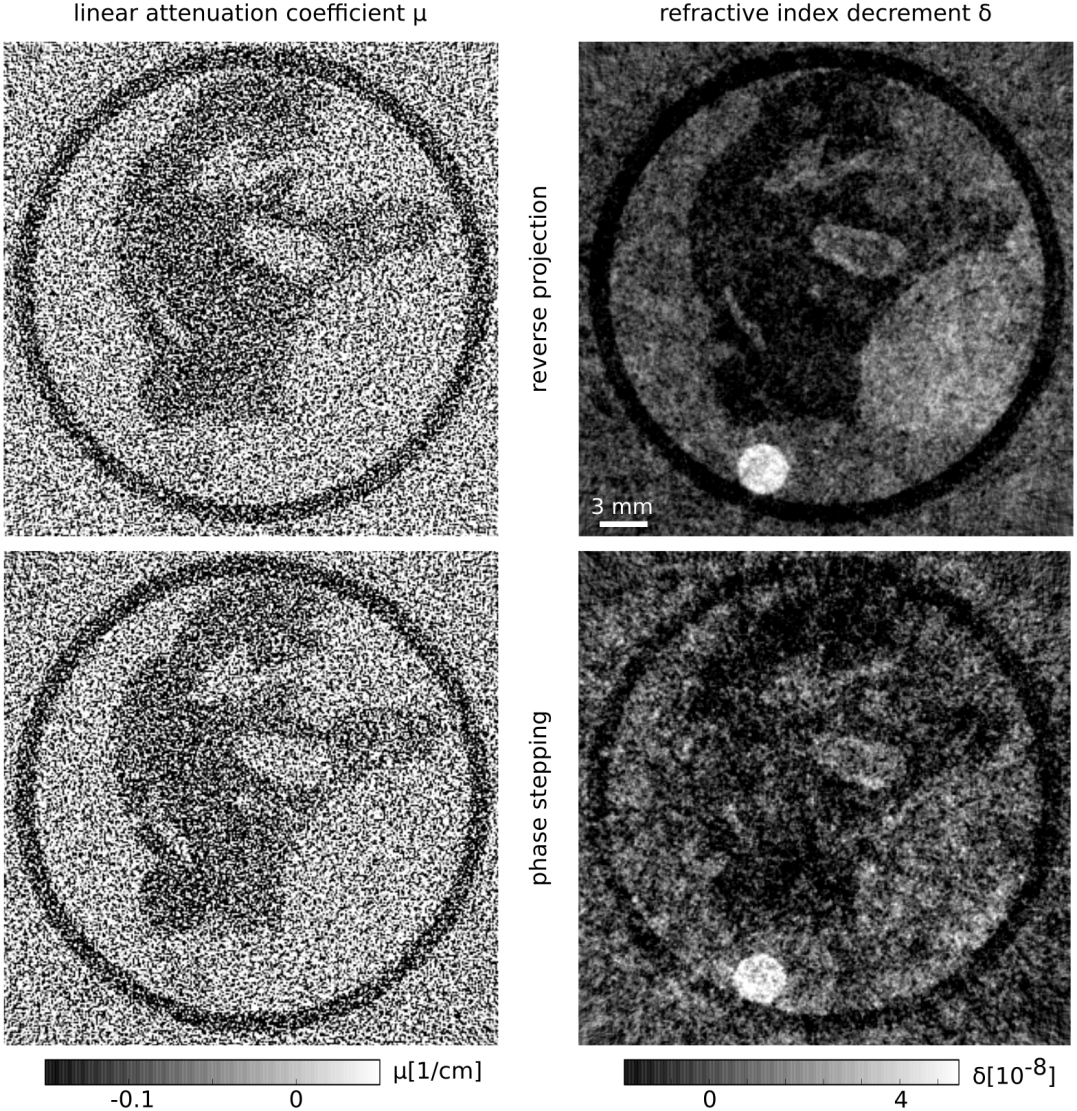
Comparison of reconstructions obtained with the RP method (top, 70 counts/pixel/projection) and the PS approach (bottom, 76 counts/pixel/projection). Note, that while the total applied dose is even slightly lower for the scan obtained with the RP method, the reconstruction of the refractive index decrement still shows much better image quality and also less noise compared to the PS method. As expected, there is no visible difference in the reconstruction of the linear attenuation coefficient.

The comparison is displayed in Fig 6. Evidently, the reconstruction of the linear attenuation coefficient has the same visual appearance for both methods. This is an expected result, since both methods simply average all phase steps for the retrieval of the attenuation projections. However, the situation looks quite different in the phase-contrast channel, where the reconstruction of the RP projections has superior image quality. There is less noise and thus more features can be recognized compared to the PS reconstruction. This can be explained by the lower amount of noise that is present in the DPC projections of the RP method. Additionally, the RP projections are not affected by statistical phase wrapping and its corresponding loss of signal in some pixels. This effect not only worsens the image quality of the PS reconstruction but also affects its quantitativeness. This is evident from the comparison of the mean values of the three homogeneous regions described above to the values of the reference scan. The results of this analysis are displayed in rows 3 and 4 of Table 1. Clearly, the RP method delivers more accurate values of the refractive index decrement. For all three materials, the relative errors lie between −6 and 7 percent. In contrast, the relative errors in the PS reconstructions are between −17 and −31 percent. It is evident that the *δ* values are severely underestimated, suggesting statistical phase wrapping as the cause: In pixels affected by this phenomenon, the phase is not retrieved correctly. Instead, random values from a uniform distribution, which correspond to a reconstructed mean *δ* of zero, are returned. Therefore, non-significant amounts of phase-wrapped pixels lead to mean values closer to zero.

The RMSE and SSI values of the two reconstructions, compared to the reference scan, are displayed in rows 1 and 2 of Table 2. Here, too, the RP methods shows better performance, namely a lower RMSE and a higher SSI in comparison to the PS approach.

**Table 2:** Root mean squared error and structure similarity of the tomographic reconstructions displayed in Figs 6 and 7 compared to the reference scan.

scan descr.	RMSE [⋅10^−8^]	SSI
Fig 6		
PS (76 cts)	1.34	0.89
RP (70 cts)	0.89	0.95
Fig 7		
PS (111 cts)	1.26	0.89
RP (44 cts)	1.03	0.93

**Fig 7:**
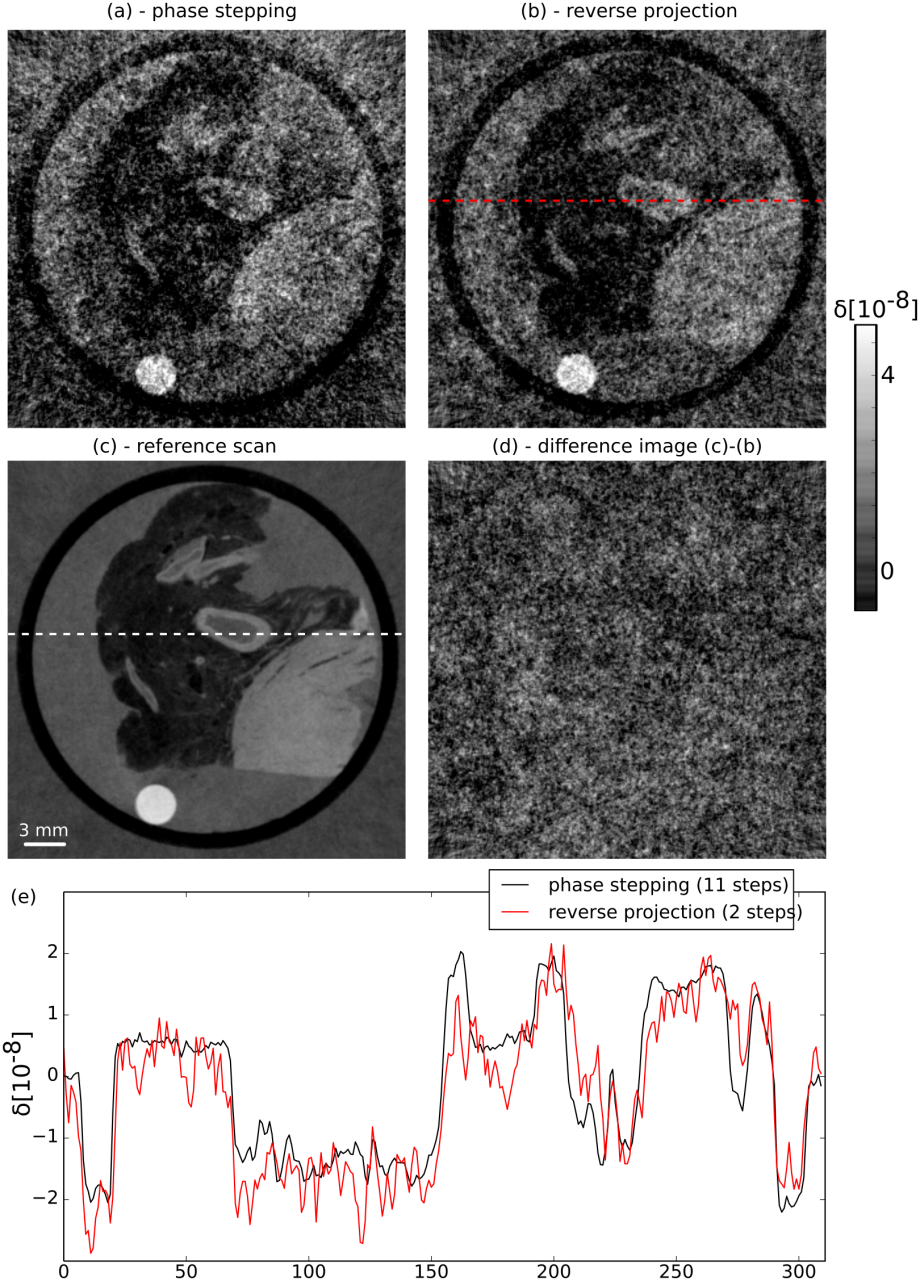
Image quality and quantitative accuracy of the RP method in a low-dose scenario. Comparison of the reconstructions obtained with the PS method (a, 111 counts/pixel/projection) and the RP method (b, 44.4 mean counts) with a reference scan (c). Remarkably, the RP reconstruction shows superior image quality compared to the PS method, even though only 2 of the 5 steps of the PS approach are used to generate the RP reconstruction. Panel (d) shows a difference image of (b) and (c), which is dominated by noise. That implies a good quantitative accuracy of the RP method. This finding is confirmed by line plot in panel (e) which shows plots along the lines displayed in panels (b) and (c). Note that the values for the RP method were averaged over 4 slices and 4 pixels in direction perpendicular to the line for improved readability.

It could be argued that the superior quality of the RP reconstructions stems solely from the lower noise level in its projections compared to the PS projections, as the noise level is lower for the RP approach when using the same exposure time for both methods. To dispute this assumption and examine more closely the influence of statistical phase wrapping, we have compared two scans that were obtained using the same set of phase stepping images. In that case, both scans have the same number of mean photon counts per phase step, namely 22.2 counts. As before, five phase stepping images are used to obtain the DPC projections for the PS method, leading to a mean total number of 111 counts per pixel and projection. In contrast, only two steps are used to extract the DPC projections in the RP method, amounting to 44.4 counts per pixel and projection. That means the number of counts per pixel is higher by a factor of 2.5 for the PS method (cf. Fig 5). Consequently, one would expect that the tomographic reconstruction of the PS projections features superior image quality. However, when looking at these reconstructions (Fig 7a and 7b), it can be clearly seen that this is not at all the case. The reconstruction of the PS projections exhibits more noise than the reconstruction of the RP projections. Thus, the image quality of the RP reconstruction is superior, which also leads to better feature recognition in this image. These results show that statistical phase wrapping in the projections has a drastic negative effect on the image quality in the reconstructions. Additionally, statistical phase wrapping leads to wrong quantitative values, which will be investigated next.

First, we visually compare the reconstructed values of the RP method with the reference scan using a difference image, which is displayed in Fig 7(d). This image is dominated by noise and there are no features visible, suggesting that the RP method is able to quantitatively reconstruct the refractive index decrement even at very low photon counts. Second, a line plot (Fig 7(e)) of the same two images is used for further comparison. Note that the line of the RP scan was obtained by averaging over 4 slices and 4 pixels in direction perpendicular to the line in order to decrease the noise level and thereby make the interpretation of the plot easier. The line corresponding to the RP reconstruction matches the reference line quite well, although there are still fluctuations due to noise despite the averaging procedure. Overall, the quantitativeness is comparable to the analysis that was performed in Fig 3. Next, the mean *δ* values that were measured in the three homogeneous regions are inspected. The values are shown in rows 5 and 6 of Table 1. For all three materials, the values obtained by the RP method are closer to the reference values than the PS values. Additionally, the standard deviation in the three regions is also lower for the RP method. Finally, a comparison of RMSE and SSI of the two reconstructions (Table 2, rows 3 and 4) also shows superior performance of the RP method. Overall, these finding are quite remarkable: discarding three of the five phase steps, and with that 60% of the photons, results in superior image quality and more accurate quantitative values in the tomographic reconstruction.

## Discussion

In the previous section, we have shown an improved performance of the RP method compared to the conventional PS method in low-dose applications. This improved performance essentially stems from the use of prior knowledge in the phase retrieval: the dynamic range is (artificially) limited compared to the conventional approach, which can be justified by the prevalence of small values in a differential signal.

Evidently, there are also limitations to this method. First, the phase retrieval is based on an approximation that is only correct for small values of the phase shift. On the one hand, most of the values typically are small due to the differential nature of the phase-contrast signal. On the other hand, the influence of large values is disproportionately high due to the necessary integration during the filter step of the tomographic reconstruction.

Secondly, only the attenuation and the phase-contrast signal can be obtained. The dark-field signal is not only inaccessible, but even leads to wrongly determined phase shift values. Both facts limit the applicability of the RP method to certain types of samples in order to achieve satisfactory quantitative reconstructions. However, one could imagine extensions to this method that could alleviate some of these issues. Among them is the possibility of employing a third phase step to quantify the dark-field signal [36].

Additionally, the straight forward experimental application of the method is limited to cases where the reference phase is uniform over the whole field of view. This is a result of grating imperfections which could be solved by future, more uniform gratings from improved fabrication processes. As a uniform reference phase was not available for our experiment, we had to record more than the two theoretically necessary phase steps and then retrospectively select the appropriate steps for each pixel separately. With the 11 phase steps that were recorded in our experiment, a step close to the optimal point for phase retrieval was available for each pixel. Thus, we could simply employ a linear correction for the remaining deviation from the optimal point.

By using a higher order function instead of a simple linear approximation for phase retrieval, the phase shift could also be calculated accurately with points farther away from the linear region. Consequently, fewer steps would be needed to achieve successful phase retrieval even when the reference phase is not uniform over the field of view. However, more than two phase steps would still be required, since meaningful phase retrieval with only two points is not possible when they lie around the turning points of the phase stepping curve. If e.g. four equidistant phase steps were recorded, there would always be at least two steps (or combinations of steps) that could be used for the RP phase retrieval. In this case, the RP approach would still lead to superior reconstructions in low dose scans as was demonstrated in the example displayed in Fig 6, where even five phase steps were used for the conventional phase retrieval. In a high statistic scan, the noise levels would be similar in both approaches (cf. Eqs (1) and (11)).

Such an advanced scheme could also be combined with iterative reconstruction (IR) methods including algebraic iterative reconstruction and statistical iterative reconstruction [37–40]. In particular with statistical iterative reconstruction (SIR) schemes, where the pixels could be assigned a weight corresponding to their noise levels [41]. In general, SIR can be used to significantly increase the image quality of tomographic reconstructions. However, it is important to note that conventional iterative reconstruction schemes work on the already retrieved projections. If a wrong phase is retrieved (e.g. in the PS approach due to statistical phase wrapping), the IR’s ability to improve the image quality is hindered. It can not correct for the influence of “wrong pixels” because there is no way to know which pixels are affected. In the RP approach, where the phase is determined correctly and only affected by Gaussian noise, SIR schemes will be more successful in improving the image quality of the reconstructions. Thus, studying the effect of SIR for phase-contrast CT on the image noise in a low dose regime might be subject of further work.

Lately, intensity-based iterative reconstruction schemes for differential phase contrast data have been introduced [42–44], where the reconstruction is done directly from the measured intensities. This means that the intermediate step of phase retrieval is directly incorporated into the tomographic reconstruction. In this case, the prior knowledge of small differential phase shifts has to be incorporated in a different way (e.g. via regularization). Whether these methods can achieve meaningful phase retrieval at low photon counts has yet to be shown.

## Conclusion

Up until now, it has been assumed that the potential for dose reduction in phase-contrast CT is limited compared to attenuation CT. That is one obstacle that stands in the way of more mainstream or even clinical application of phase-contrast CT. This claim is mainly based on the work of Raupach & Flohr (2011) [22], where a low-dose limit for phase-contrast CT is derived. However, only the phase stepping approach for phase retrieval is considered in their work. As we have shown here, a phase stepping approach is not the optimal choice for scans with low mean photon counts, since statistical phase wrapping leads to adverse effects on image quality. As a possible alternative, we have examined the reverse projection method and have illustrated that it yields quantitatively and visually satisfactory results for phase-contrast CT scans of biological soft-tissue. More importantly, our results also show that this method can extract the differential phase correctly where phase retrieval via phase stepping fails due to statistical phase wrapping. However, there are limitations to this method currently still standing in the way of a widespread application and more development is necessary to tackle these challenges. Overall, we imagine that based on the results of this study further phase retrieval and reconstruction schemes can be developed that are optimized for low-dose applications.

## References

[1] Bonse U, Hart M. An X-ray interferometer. Appl Phys Lett. 1965;6(1965):155–156. 10.1063/1.1754212

[2] Momose A, Takeda T, Itai Y, Hirano K. Phase-contrast X-ray computed tomography for observing biological soft tissues. Nat Med. 1996;2(4):473–475. 10.1038/nm0496-473 8597962

[3] David C, Nöhammer B, Solak HH, Ziegler E. Differential x-ray phase contrast imaging using a shearing interferometer. Appl Phys Lett. 2002;81(17):3287 10.1063/1.1516611

[4] Weitkamp T, Diaz A, David C, Pfeiffer F, Stampanoni M, Cloetens P, et al X-ray phase imaging with a grating interferometer. Opt Express. 2005;13(16):6296–304. 10.1364/OPEX.13.006296 19498642

[5] Förster E, Goetz K, Zaumseil P. Double crystal diffractometry for the characterization of targets for laser fusion experiments. Krist und Tech. 1980;15(8):937–945. 10.1002/crat.19800150812

[6] Davis TJ, Gao D, Gureyev TE, Stevenson aW, Wilkins SW. Phase-contrast imaging of weakly absorbing materials using hard X-rays. Nature. 1995;373(6515):595–598. 10.1038/373595a0

[7] Olivo A, Speller R. A coded-aperture technique allowing x-ray phase contrast imaging with conventional sources. Appl Phys Lett. 2007;91(7):1–3. 10.1063/1.2772193

[8] Momose A. Recent Advances in X-ray Phase Imaging. Jpn J Appl Phys. 2005;44(9A):6355–6367. 10.1143/JJAP.44.6355

[9] Pfeiffer F, Weitkamp T, Bunk O, David C. Phase retrieval and differential phase-contrast imaging with low-brilliance X-ray sources. Nat Phys. 2006;2(4):258–261. 10.1038/nphys265

[10] Pfeiffer F, Kottler C, Bunk O, David C. Hard X-Ray Phase Tomography with Low-Brilliance Sources. Phys Rev Lett. 2007;98(10):108105 10.1103/PhysRevLett.98.108105 17358572

[11] Pfeiffer F, Bech M, Bunk O, Kraft P, Eikenberry EF, Brönnimann C, et al Hard-X-ray dark-field imaging using a grating interferometer. Nat Mater. 2008;7(2):134–137. 10.1038/nmat2096 18204454

[12] Bravin A, Coan P, Suortti P. X-ray phase-contrast imaging: from pre-clinical applications towards clinics. Phys Med Biol. 2013;58(1):R1 10.1088/0031-9155/58/1/R1 23220766

[13] Pfeiffer F, Herzen J, Willner M, Chabior M, Auweter S, Reiser M, et al Grating-based X-ray phase contrast for biomedical imaging applications. Z Med Phys. 2013;23(3):176–85. 10.1016/j.zemedi.2013.02.002 23453793

[14] Bech M, Tapfer A, Velroyen A, Yaroshenko A, Pauwels B, Hostens J, et al In-vivo dark-field and phase-contrast x-ray imaging. Sci Rep. 2013;3:10–12. 10.1038/srep03209 PMC3826096 24220606

[15] Velroyen A, Yaroshenko A, Hahn D, Fehringer A, Tapfer A, Müller M, et al. Grating-based X-ray Dark-field Computed Tomography of Living Mice. EBioMedicine. 2015; 10.1016/j.ebiom.2015.08.014 PMC4634200 26629545

[16] Roessl E, Daerr H, Koehler T, Martens G, van Stevendaal U. Clinical boundary conditions for grating-based differential phase-contrast mammography. Philos Trans R Soc A Math Phys Eng Sci. 2014;372 (2010). 10.1098/rsta.2013.0033 24470415

[17] Koehler T, Daerr H, Martens G, Kuhn N, Löscher S, van Stevendaal U, et al Slit-scanning differential x-ray phase-contrast mammography: Proof-of-concept experimental studies. Med Phys. 2015;42(4):1959–1965. 10.1118/1.4914420 25832086

[18] Noël PB, Fingerle AA, Renger B, Munzel D, Rummeny EJ, Dobritz M. Initial Performance Characterization of a Clinical Noise-Suppressing Reconstruction Algorithm for MDCT. Am J Roentgenol. 2011;197(6):1404–1409. 10.2214/AJR.11.6907 22109296

[19] Noël PB, Renger B, Fiebich M, Münzel D, Fingerle Aa, Rummeny EJ, et al Does iterative reconstruction lower CT radiation dose: Evaluation of 15,000 examinations. PLoS One. 2013;8(11):1–7. 10.1371/journal.pone.0081141 PMC3841128 24303035

[20] Schuhbaeck A, Achenbach S, Layritz C, Eisentopf J, Hecker F, Pflederer T, et al Image quality of ultra-low radiation exposure coronary CT angiography with an effective dose <0.1 mSv using high-pitch spiral acquisition and raw data-based iterative reconstruction. Eur Radiol. 2013;23(3):597–606. 10.1007/s00330-012-2656-2 22983283

[21] Deák Z, Grimm JM, Treitl M, Geyer LL, Linsenmaier U, Körner M, et al Filtered back projection, adaptive statistical iterative reconstruction, and a model-based iterative reconstruction in abdominal CT: an experimental clinical study. Radiology. 2013;266(1):197–206. 10.1148/radiol.12112707 23169793

[22] Raupach R, Flohr TG. Analytical evaluation of the signal and noise propagation in x-ray differential phase-contrast computed tomography. Phys Med Biol. 2011;56(7):2219–44. 10.1088/0031-9155/56/7/020 21403187

[23] Wang M, Zhu PP, Zhang K, Hu XF, Huang WX, Cen YW, et al A new method to extract angle of refraction in diffraction enhanced imaging computed tomography. J Phys D Appl Phys. 2007;40(22):6917–6921. 10.1088/0022-3727/40/22/010

[24] Zhu P, Zhang K, Wang Z, Liu Y, Liu X, Wu Z, et al Low-dose, simple, and fast grating-based X-ray phase-contrast imaging. Proc Natl Acad Sci U S A. 2010;107(31):13576–13581. 10.1073/pnas.1003198107 20643971 PMC2922255

[25] Wu Z, Wang ZL, Gao K, Wang DJ, Wang SH, Chen J, et al Signal-to-noise ratio analysis of X-ray grating interferometry with the reverse projection extraction method. J Electron Spectros Relat Phenomena. 2014;196:75–79. 10.1016/j.elspec.2014.04.011

[26] Wu Z, Gao K, Chen J, Wang D, Wang S, Chen H, et al High sensitivity phase retrieval method in grating-based x-ray phase contrast imaging. Med Phys. 2015;42(2):741–49. 10.1118/1.4905490 25652488

[27] Revol V, Kottler C, Kaufmann R, Straumann U, Urban C. Noise analysis of grating-based x-ray differential phase contrast imaging. Rev Sci Instrum. 2010;81(7):073709 10.1063/1.3465334 20687733

[28] Weber T, Bartl P, Bayer F, Durst J, Haas W, Michel T, et al Noise in x-ray grating-based phase-contrast imaging. Med Phys. 2011;38(2011):4133–4140. 10.1118/1.3592935 21859014

[29] Chen GH, Zambelli J, Li K, Bevins N, Qi Z. Scaling law for noise variance and spatial resolution in differential phase contrast computed tomography. Med Phys. 2011;38(2):584 10.1118/1.3533718 21452695 PMC3030613

[30] Wang ZL, Gao K, Chen J, Ge X, Zhu PP, Tian YC, et al A new method for information retrieval in two-dimensional grating-based X-ray phase contrast imaging. Chinese Phys B. 2012;21(11):118703 10.1088/1674-1056/21/11/118703

[31] Wu Z, Gao K, Wang Z, Ge X, Chen J, Wang D, et al A new method to retrieve phase information for equiangular fan beam differential phase contrast computed tomography. Med Phys. 2013;40(2013):031911 10.1118/1.4791672 23464326

[32] Wang Z, Gao K, Wang D. Phase retrieval with the reverse projection method in the presence of object’s scattering. Radiat Phys Chem. 2015;(February).

[33] Birnbacher L, Willner M, Velroyen A, Marschner M, Hipp A, Meiser J, et al Experimental Realisation of High-sensitivity Laboratory X-ray Grating-based Phase-contrast Computed Tomography. Sci Rep. 2016;6:24022 10.1038/srep24022 27040492 PMC4819174

[34] Zanette I, Weitkamp T, Lang S, Langer M, Mohr J, David C, et al Quantitative phase and absorption tomography with an X-ray grating interferometer and synchrotron radiation. Phys Status Solidi. 2011;208(11):2526–2532. 10.1002/pssa.201184276

[35] Willner M, Herzen J, Grandl S, Auweter S, Mayr D, Hipp A, et al Quantitative breast tissue characterization using grating-based x-ray phase-contrast imaging. Phys Med Biol. 2014;59(7):1557–71. 10.1088/0031-9155/59/7/1557 24614413

[36] Pelliccia D, Rigon L, Arfelli F, Menk RH, Bukreeva I, Cedola A. A three-image algorithm for hard x-ray grating interferometry. Opt Express. 2013;21(16):19401–11. 10.1364/OE.21.019401 23938856

[37] Fu J, Hu X, Velroyen A, Bech M, Jiang M, Pfeiffer F. 3D algebraic iterative reconstruction for cone-beam X-Ray differential phase-contrast computed tomography. PLoS One. 2015;10(3):1–13. 10.1371/journal.pone.0117502 PMC4361763 25775480

[38] Fu J, Velroyen A, Tan R, Zhang J, Chen L, Tapfer A, et al A reconstruction method for cone-beam differential x-ray phase-contrast computed tomography. Opt Express. 2012;20(19):21512–9. 10.1364/OE.20.021512 23037271

[39] Koehler T, Brendel B, Roessl E. Iterative reconstruction for differential phase contrast imaging using spherically symmetric basis functions. Med Phys. 2011;38(8):4542 10.1118/1.3608906 21928625

[40] Gaass T, Potdevin G, Bech M, Noël PB, Willner M, Tapfer A, et al Iterative reconstruction for few-view grating-based phase-contrast CT—An in vitro mouse model. EPL (Europhysics Lett. 2013;102(4):48001 1–6.

[41] Hahn D, Thibault P, Fehringer A, Bech M, Koehler T, Pfeiffer F, et al Statistical iterative reconstruction algorithm for X-ray phase-contrast CT. Sci Rep. 2015;5:10452 10.1038/srep10452 26067714 PMC4464273

[42] Ritter A, Bayer F, Durst J, Gödel K, Haas W, Michel T, et al Simultaneous maximum-likelihood reconstruction for x-ray grating based phase-contrast tomography avoiding intermediate phase retrieval. ArXiv e-prints. 2013; p. 1–5.

[43] Brendel B, von Teuffenbach M, Fehringer A, Noël PB, Pfeiffer F, Koehler T. Intensity-Based Iterative Reconstruction for Differential Phase-Contrast Imaging with Reconstruction Parameter Estimation. In: Proc. 13th Int. Meet. Fully Three-Dimensional Image Reconstr. Radiol. Nucl. Med.; 2015. p. 713–716.

[44] Brendel B, von Teuffenbach M, Noël PB, Pfeiffer F, Koehler T. Penalized maximum likelihood reconstruction for x-ray differential phase-contrast tomography. Med Phys. 2016;43(1):188–194. 10.1118/1.4938067 26745911

